# Impact of a pharmacy resident on a transitions of care rotation for inpatients enrolled in an outpatient parenteral antimicrobial therapy (OPAT) program

**DOI:** 10.1017/ash.2023.181

**Published:** 2023-06-29

**Authors:** Rachel S. Britt, Jeffrey C. Pearson, Mary T. LaSalvia, Monica V. Mahoney, Christopher McCoy, Simi Padival

**Affiliations:** 1 Department of Pharmacy, The University of Texas Medical Branch at Galveston, Galveston, Texas; 2 Department of Pharmacy, Brigham & Women’s Hospital, Boston, Massachusetts; 3 Division of Infectious Diseases, Beth Israel Deaconess Medical Center, Boston, Massachusetts; 4 Department of Pharmacy, Beth Israel Deaconess Medical Center, Boston, Massachusetts; 5 Division of Infectious Diseases, University of Pittsburgh Medical Center, Pittsburgh, Pennsylvania

## Abstract

A novel pharmacy residency rotation was created to meet the needs of patients enrolled in an outpatient parenteral antimicrobial therapy (OPAT) program but not yet discharged from the inpatient setting. This service resulted in a high number of antimicrobial stewardship interventions identified and accepted by the primary team(s).

Outpatient parenteral antimicrobial therapy (OPAT) is defined as the administration of at least 2 doses of intravenous antimicrobial agents outside the acute-care setting on 2 different calendar days.^1^ More than 250,000 patients are estimated to receive OPAT services annually in the United States.^1,2^ Additionally, more data are demonstrating the noninferiority of oral therapy compared to intravenous, and more institutions are incorporating longer-term oral or partial-oral therapy into their OPAT programs.^3–6^ Pharmacists are well poised to be integrated into OPAT programs.^7,8^

Since 2010, the American Society of Health-System Pharmacists (ASHP) has advocated for the Practice Advancement Initiative (PAI), to drive pharmacy practice change at the local level.^9^ PAI 2030 consists of 59 recommendations to promote optimal, safe, and effective medication use and to ensure that the pharmacy profession is able to meet the demands of future practice and patient-care delivery models.^10^

At Beth Israel Deaconess Medical Center (BIDMC), a new post-graduate year 2 (PGY2) infectious diseases (ID) OPAT rotation was developed. The reasons for this were 2-fold: (1) to offer a new rotational experience and (2) to provide additional pharmacologic oversight before a formal, dedicated, OPAT pharmacist practice was available. At the time of rotation creation, the BIDMC OPAT program consisted of an attending ID physician, a part-time program coordinator, and ID nurses. Patients who were discharged from BIDMC on at least 2 weeks of intravenous antimicrobial therapy were eligible for enrollment in the OPAT program. The BIDMC program follows patients at all sites of care postdischarge including home, infusion center, and skilled nursing facilities. The site of care was determined in coordination with the primary team and case management team. Patients were required to be seen by the ID service prior to discharge for formal OPAT enrollment to ensure that the patient was clinically appropriate for outpatient parenteral therapy.^1^ Once enrolled, a structured note was placed into the electronic health record outlining the infection type, discharge antimicrobial agent(s), OPAT start date, anticipated OPAT end date, and weekly safety laboratory tests. When a patient was enrolled in OPAT, the inpatient ID service signed the patient out and added the patient to the OPAT patient dashboard. The dashboard contained high-level information such as patient name, medical record number, infection type, date(s) of enrollment and hospital discharge, discharge location, and due date for next laboratory test results. The ID service did not regularly see the patients during their remaining inpatient stay unless the primary team alerted them to a clinical status change. As such, there are frequently intervals of time between OPAT enrollment and hospital discharge while awaiting care coordination or necessary clinical improvement for discharge.

A new PGY2-ID pharmacy-resident rotation in OPAT was created at BIDMC in 2018. It consisted of dedicated PGY2-ID pharmacy-resident time, Monday through Friday during the day shift, for a period of 4 weeks. The pharmacy resident reviewed the OPAT dashboard for patients newly enrolled in the OPAT program but still admitted at the institution. Daily activities involved reviewing patient clinical status and laboratory results, microbiologic information, antimicrobial selection, dosing, duration, route, and allergy information. Key areas for review included adverse reactions not limited to hypersensitivity reactions, cytopenia, liver function test (LFT) abnormalities, altered mental status, and change in renal function. Additionally, the pharmacy resident reviewed any new culture data for further de-escalation opportunities and development of new infections. The pharmacy resident met with patients at the bedside prior to discharge, introduced the OPAT program, counsel the patient and/or their caregiver(s) on the antimicrobial(s), and made adjustment recommendations to the primary and/or OPAT team when needed.

Two PGY2-ID pharmacy residents completed the rotation (April 23, 2018–May 11, 2018 and March 11, 2019–April 12, 2019). Patient encounters and recommendations were documented in the electronic health record and were communicated to the primary and/or OPAT team(s). Interventions were also entered in a standalone Microsoft Excel database (Microsoft, Redmond, WA). This review was deemed exempt from approval by the BIDMC Institutional Review Board. This rotation was featured as a PAI 2030 case study on the ASHP website.^11^

During the combined 8-week period, 109 patients were enrolled in OPAT and were eligible for study inclusion. Among them, 77 patients (70.6%) were reviewed by the pharmacy residents. The main reason for lack of review was quick and/or weekend discharge after OPAT enrollment. Demographics are presented in Table 1. Most patients were male (57%), with a mean age of 62.6 years (range, 21–96). The most common infectious indications for OPAT were osteomyelitis (29.0%), bacteremia (28.0%), and abscess (10.8%).

**Table 1.** Patient Demographics

Characteristic	Result, No. (%)^a^
Age, mean y (range)	62.6 (21–96)
Sex, male	44 (57.1)
**Infection type^b^**	N=94
Osteomyelitis	27 (28.7)
Bacteremia	27 (28.7)
Abscess	10 (10.6)
Endocarditis	7 (7.4)
Skin/soft tissue or wound	4 (4.3)
Prosthetic joint infection	3 (3.2)
Intra-abdominal infection	3 (3.2)
Other^c^	13 (13.9)
**No. of discharge antimicrobial(s)**	
1 antimicrobial	43 (33.8)
2 antimicrobials	26 (33.8)
3 antimicrobials	8 (10.4)
**Discharge antimicrobial**	N=111
Vancomycin	22 (28.6)
Ceftriaxone	16 (20.8)
Cefazolin	16 (20.8)
Daptomycin	11 (14.3)
Metronidazole	9 (11.7)
Meropenem	6 (7.8)
Cefepime	6 (7.8)
Nafcillin	5 (6.5)
Other^d^	20 (18.2)
**No. of intervention(s) made per patient**	
0 interventions	28 (36.4)
1 intervention	39 (50.6)
2 interventions	6 (7.8)
3 interventions	4 (5.2)

a No. (%) unless otherwise indicated.

b 15 patients had ≥1 infection each: 2 infections (n=13), 3 infections (n=3).

c Other infection types: empyema (n=2), urinary tract infection (n=2), diabetic foot infection (n=2), endovascular (n=2), pneumonia (n=2), septic joint infection (n=2), encephalitis (n=1).

d Other antimicrobials: ertapenem (n=3), ampicillin (n=2), ceftolozane-tazobactam (n=2), ciprofloxacin (n=2), fluconazole (n=2), piperacillin/tazobactam (n=2), rifampin (n=2), acyclovir (n=1), cefpodoxime (n=1), gentamicin (n=1), levofloxacin (n=1), linezolid (n=1).

Most patients were discharged on 1 antimicrobial agent (55.8%), although 33.8% were discharged on 2 agents, and 10.4% were discharged on 3 agents. The most common antimicrobials upon discharge included vancomycin (28.6%), ceftriaxone (20.6%), cefazolin (20.8%), daptomycin (14.3%), metronidazole (11.7%), cefepime (7.8%), and meropenem (7.8%).

The pharmacy residents performed a total of 85 patient visits (range, 0–5 visits per patient). Overall, 63 interventions were recommended, with 50 (79.3%) accepted by the team(s). Most patients received 1 intervention; however, that number ranged from 0 to 3 interventions per patient. The most common interventions included dosing recommendations (23.8%), coordination of care (20.6%), antibiotic choice (12.7%), monitoring (12.7%), and duration of therapy (7.9%) (Fig. 1). Moreover, 55 interventions involved medications; the most common were intravenous vancomycin (25.5%), daptomycin (21.8%), ceftriaxone (10.9%), cefazolin (9.1%), and metronidazole (7.3%) (Fig. 2). During this time, vancomycin monitoring was trough-based. The most frequent infection types intervened on included osteomyelitis (32.5%), bacteremia (27.3%), endocarditis (9.1%), and abscesses (4.3%).

**Figure 1.**
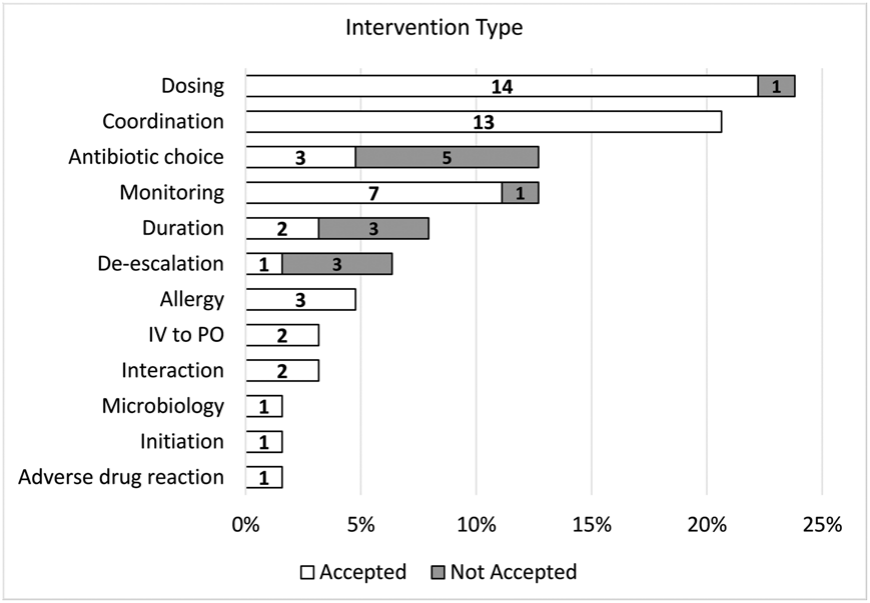
OPAT intervention types recommended by pharmacy residents. Note: Abx, antimicrobial; ADR, adverse drug reaction; IV, intravenous; OPAT, outpatient parenteral antimicrobial therapy; PO, per os (ie, oral). Numbers within the bars represent individual number of interventions recommended.

**Figure 2.**
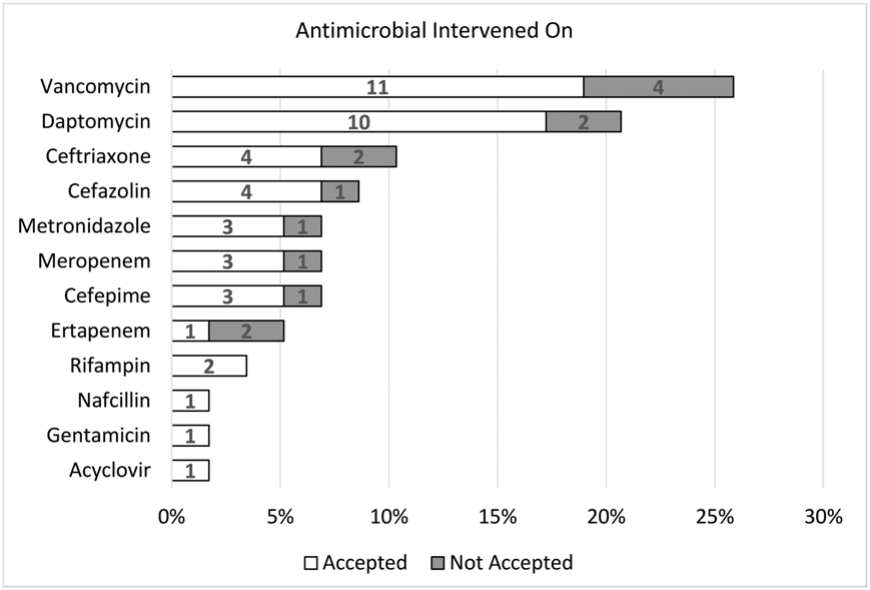
Antimicrobial agents that received pharmacy-resident interventions. Numbers within the bars represent individual number of interventions recommended.

Compared to the ID team, the pharmacy resident was able to visit the patient closer to discharge for 61 patients (79.2%). Overall, the ID team saw the patient a median of 3 days (interquartile range [IQR], 1–9.5) prior to discharge, compared to the pharmacy resident seeing the patient 1 day (IQR, 0–4) prior to discharge. Additionally, the ID team was able to see 31 patients (40.3%) within 1 day of discharge, compared to the pharmacy resident seeing 44 patients (57.1%) within 1 day of discharge.

OPAT is an emerging area of practice for pharmacists, and there are many opportunities for intervention.^7,8^ Our experience highlights 2 distinct 4-week periods in which pharmacy residents were proactive in their activities. The pharmacy residents helped fill gaps in care, such as the ability to provide an additional focused review reinforcing patient–provider communication just prior to discharge, which was not previously provided by any healthcare provider prior to this rotation experience. These additional reviews allowed the pharmacy resident a more accurate and updated view of clinical status, resulting in individualized and optimized care. Additionally, the pharmacy residents were able to speak directly to the patient and/or caregivers, providing education and reinforcement of knowledge. Drug dosing was the most common intervention type, but this was followed closely by coordination of care. Examples of coordination of care included liaising between care teams to discuss patient concerns or updates in care plans and scheduling additional follow-up appointments or counseling sessions.

Both OPAT pharmacy practice and pharmacy-resident OPAT rotations can meet several PAI 2030 initiatives.^10^ Speaking with patients and/or caregivers can help educate them on the antimicrobial agents and administration across the continuum of care. Expanding the pharmacist role into OPAT and creating pharmacy-resident rotation opportunities can help develop new service lines and practices. Due to its success, this experience continues to be offered to our pharmacy residents and helped to establish the need for a full-time OPAT pharmacist. Although this rotation focused on pharmacy residents, core elements of the intervention types could also be tailored to nursing, physician, and other healthcare trainees.

In conclusion, establishing a pharmacy-resident rotation on an OPAT service resulted in a high number of interventions identified and accepted by the primary team(s). The interval between OPAT enrollment and patient discharge presents opportunity to maximize antimicrobial stewardship principles.
